# Sustainable Fortification of Corn Tortillas with Broccoli By-Products

**DOI:** 10.3390/foods14050799

**Published:** 2025-02-26

**Authors:** Nieves García-Lorca, Concetta Libero, Carmela Livigni, Natalia Eleftheria Frouzaki, Encarna Aguayo

**Affiliations:** 1 Postharvest and Refrigeration Group, Universidad Politécnica de Cartagena (UPCT), Paseo Alfonso XIII, 48, 30203 Cartagena, Spain; nieves.garcial@upct.es; 2 Food Quality and Health Group, Institute of Plant Biotechnology (IBV-UPCT), Campus Muralla Del Mar, 30202 Cartagena, Spain; 3 Faculty of Agriculture, University of Naples “Federico II” via Università, 100-80055 Portici, 80055 Naples, Italy; concetta.libero97@gmail.com (C.L.); carmen.livigni100514@gmail.com (C.L.); 4 Department of Food Science and Nutrition, School of Environment, University of the Aegean, Metropolite Ioakeim 2, 81400 Myrina, Lemnos, Greece; nataliafrouzaki78@gmail.com

**Keywords:** bioactive compounds, glucosinolates, neoglucobrassicin, minerals, functional ingredients, bakery, zero hunger

## Abstract

Fortification is the deliberate addition of essential micronutrients, such as vitamins and minerals, to enhance a food’s nutritional profile and contribute to public health. A promising approach to fortification involves the use of plant by-products which are rich in bioactive compounds. This study evaluates the effects of incorporating broccoli by-product powder into corn-flour tortillas. Five formulations were developed: a control (100% corn flour) and variations replacing 2.5%, 5%, 7.5%, and 10% of the corn flour with broccoli by-product powder. Adding broccoli powder resulted in darker tortillas with slightly reduced firmness. Water and oil absorption capacities increased in fortified tortillas compared to the control. The broccoli powder in the tortillas significantly enhanced their nutritional profile. Calcium content increased nearly six-fold, while potassium and iron concentrations were tripled in tortillas fortified with 10% broccoli powder. Additionally, dietary fiber content rose by 23%. Antioxidant capacity improved significantly, particularly in total polyphenol content. Fortification also led to a significantly higher glucosinolate concentrations, notably neoglucobrassicin and glucoraphanin. Sensory evaluation showed that consumers found tortillas containing 2.5% to 7.5% broccoli powder to be the most acceptable. However, fortification at 10% negatively impacted overall acceptability, primarily due to the intensified brassica flavor. In conclusion, incorporating broccoli by-product powder into corn tortillas enhanced their nutritional and functional properties, whilst retaining acceptable sensory characteristics. This approach promotes the sustainable valorization of by-products, offering a viable, eco-friendly alternative for the development of functional, nutrient-rich foods that support sustainability in the food industry.

## 1. Introduction

The World Health Organization (WHO) defines fortification as the practice of deliberately increasing the content of an essential micronutrient, such as vitamins and minerals (including trace elements), in a food, regardless of whether the nutrients were originally present in the food before processing or not, so as to improve the nutritional quality of the food supply and provide a public health benefit with minimal risk to health [1]. In fact, the WHO recommends the inclusion of various fortified foods in all settings, such as universal salt iodization and the fortification of corn or wheat flour with vitamins and minerals. Nowadays, many people consume monotonous diets based on refined grains or ultra-processed foods, which are often high in sugars and fats but low in essential micronutrients. This lack of dietary diversity, combined with a calorie-rich but nutrient-poor food intake, contributes to nutritional deficiencies that affect overall health, the immune system, and long-term wellbeing. Recent studies have shown a significant association between the consumption of ultra-processed foods and an increased risk of mortality from different causes, especially cardiovascular disease, due to the low nutrient density and high content of potentially harmful compounds in those foods [2].

Fruits and vegetables are rich sources of essential micronutrients such as vitamins, minerals, and dietary fiber and provide a diverse range of bioactive compounds [3]. These compounds, including polyphenols, flavonoids, carotenoids, glucosinolate, etc., contribute to numerous health-promoting effects. Food-to-food fortification not only increases the nutrient content of food products, but equally importantly, the nutrient-dense fruit and vegetables used contain bioactive compounds which act as nutrient bioavailability enhancers [4]. The revalorization of by-products from the fruit and vegetable industry is gaining interest, since they represent a significant volume of discarded material which still contains valuable bioactive compounds [5] that can be utilized to improve efficiency in the food sector and promote a circular economy in the industry [6]. Agriculture produces a large proportion of biomass, and its by-products (agricultural by-products) include elements of plants and crops that are not intended for direct consumption, yet many of these can be harnessed to reduce food loss and optimize the use of critical resources such as water, land, and energy. According to the FAO [7], approximately 33% of the food produced for human consumption is wasted globally; this is equivalent to 1.3 billion tonnes per year. In this context, the processing of agricultural by-products plays a key role, as these residues from fruits, vegetables, cereals, and other raw materials contain high levels of nutrients and bioactive compounds that can be used in the manufacture of new food products and in other industrial applications [8].

The harvesting and processing of the *Brassicaceae* family, particularly genera such as broccoli and cauliflower (*Brassica oleracea*), generates a significant amount of by-products as only the florets are marketed, while the leaves and stalks are typically discarded. These by-products are rich in glucosinolates, flavonoids, and vitamins which have been shown to have antioxidant, anti-inflammatory, and anticancer properties, thus showing significant potential as candidates for enhancing the health benefits of fortified foods [9]. Therefore, the integration of *Brassica* by-products into food fortification promotes the sustainable use of agricultural resources and also enhances the bioactive compounds of fortified foods, providing a dual benefit in terms of nutrition and public health. Examples of this include tortilla chips [10], sponge cake [11], crackers [12], biscuits [13], and pizza [14].

Corn tortillas are a staple of the daily diet of Mexican people and are consumed by 94% of the population. They have had a significant cultural impact on the cuisine of southern Mexico [15]. Since 2010, tortillas have been recognized as part of the Intangible Cultural Heritage by UNESCO and were named the best street food in Latin America in 2020 [16]. The global tortilla market has experienced significant growth, driven by the popularity of Mexican cuisine and the demand for healthy options. The United States, Canada, and Mexico are the largest consumers of tortillas. In Europe, countries such as Germany, France, the United Kingdom, Italy, and Spain are increasingly consuming tortilla products. In the Asia–Pacific region, markets such as China, India, Japan, and Australia stand out for their rapid growth due to the globalization of Mexican cuisine [17]. As tortillas are widely consumed worldwide and are easy and inexpensive to produce, they are an ideal product for fortification that can also be carried out at a household scale. The addition of broccoli by-product powder to corn flour presents an innovative strategy to improve the nutritional profile of Mexican tortillas, bringing the health benefits and nutritional value of broccoli to consumers who may lack access to bioactive compounds in their diets. This study aims to evaluate the quality and sensory acceptability of tortillas enriched with various concentrations of broccoli by-product powder in the corn flour. This practical approach seeks to bring the health benefits of broccoli to individuals with unbalanced diets, whilst also promoting the circular economy by incorporating plant by-products and reducing food loss.

## 2. Materials and Methods

### 2.1. Production of Dehydrated Powder from Broccoli By-Products

Broccoli by-product powder, derived from leaves and stems, was provided by the Agrosingularity Company (Murcia, Spain). Fresh broccoli leaves and stems, collected from agricultural cooperatives in Murcia (Spain), were initially washed to remove impurities. The material was then shredded, with no predetermined particle size. Drying was carried out in a three-level belt oven using biomass (almond and walnut shells) as the heat source. The oven temperature ranged from 45 °C at the coldest point to 75 °C at the hottest, with an airspeed of 5 m/s generated by fans located at the top. The oven, which was 10 m in length, dried the material over approximately 8 h, with a belt speed of 4 m/h. Once dried, the material was ground to a particle size of 1–3 mm and subjected to pasteurization at 97–103 °C for 56 s to eliminate microorganisms. The pasteurized material was subsequently ground to a finer particle size of 0.35 mm; to preserve its properties, the temperature increase during grinding did not exceed 16 °C. Finally, the broccoli powder was packaged in 20 kg bags for sale. This process ensures that high-quality broccoli powder was produced, utilizing sustainable and efficient methods that preserved its nutritional and bioactive compounds.

### 2.2. Tortilla Preparation

The tortillas were produced following a traditional recipe. The ingredients, listed in Table 1, were mixed manually using a 0.25 mm mesh sieve to ensure homogeneity. The control dough was prepared using water, salt, and Hacendado^®^ corn flour (Mercadona, Cartagena, Spain), in the proportions shown in Table 1. Four treatments were formulated by substituting corn flour with broccoli powder at levels ranging from 2.5% to 10% of the initial weight of corn flour.

**Figure 1:**
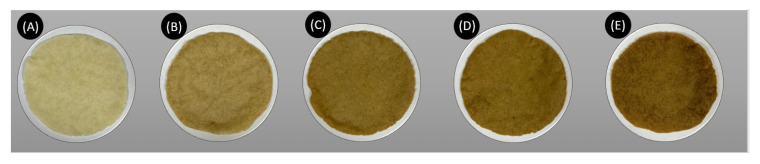
Comparison of baked corn tortillas: (**A**) Control; (**B**) tortillas fortified with 2.5% of broccoli by-product powder, (**C**) 5% (**D**) 7.5%, and (**E**) 10%.

After mixing the ingredients for each treatment, the fresh dough was divided into 40 g balls. Each ball was flattened with a 20 cm × 25 cm aluminum tortilla press (Kitchencraft, Birmingham, UK), with both metal surfaces covered with baking paper, until a disk measuring 12.9 ± 0.1 in diameter and 1.6 ± 0.05 mm in thickness was formed. The tortillas were baked in a tunnel oven (Dosilet TT3000 C regulator, Barcelona, Spain) at 300 °C for 2 min on each side with aluminum foil, followed by an additional 2 min without baking paper, making a total of 6 min. Optimal baking was assessed by observing uniform swelling across tortillas. The cooking time was recorded from the moment the tortillas were placed on the conveyor belt. The resulting baked tortillas, shown in Figure 1, measured 11.6 ± 0.1 cm in diameter and 1.6 ± 0.13 mm in thickness and weighed 30 ± 1 g. After baking, the tortillas were allowed to sit and cool at room temperature before being stored in airtight polyethylene bags. This entire experiment was repeated in its entirety on two separate occasions. All the results presented in this study come from baked tortillas.

### 2.3. Physical Properties

#### 2.3.1. Dimensions

Tortilla diameter and thickness were measured using a digital caliper (RS 200 × 0.01 mm; Mitutoyo Corporation, Kawasaki, Japan). Measurements were taken for 10 tortillas for each of the five preparations (*n* = 10).

#### 2.3.2. Color

Surface color was measured on baked tortillas and freeze-dried broccoli by-product powder using a Minolta CR-400 colorimeter (Tokyo, Japan). The device was calibrated with a white porcelain plate before use. For tortillas, measurements were obtained directly at three random points using the standard tristimulus parameters. L*, a*, and b* color values were determined using the CIELAB system. Chromaticity (C*) was calculated using Equation (1) as the square root of the sum of the squares of the a* (red-green component) and b* (yellow-blue component) values:

(1) $$C^{*}=\sqrt{a^{*2}+b^{*2}}$$

Similarly, hue angle (°h) was calculated using Equation (2), defined as the arctangent of the ratio between b* and a*:

(2) $${}^{\circ}h=arctan\frac{b^{*}}{a^{*}}$$

#### 2.3.3. Force Test

The force required to break the baked tortilla was measured using a TA-TX Plus texture analyzer (Stable Micro Systems, Godalming, UK) following the methodology described by Iuga et al. [18], with slight modifications. Circular samples were fixed between two metal plates containing a circular perforation 2.54 cm in diameter. A spherical probe (PO25S) with a 0.635 cm diameter was passed through the perforation to measure the force. The two-dimensional extensibility test was conducted in compression mode with the ‘return-to-start’ option activated. The activation force was set to 0.049 N, with pre-test, test, and post-test speeds of 10.0 mm/s, 2.0 mm/s, and 10.0 mm/s, respectively. The test continued until a distance of 30 mm was reached. Five readings were taken for each fresh tortilla, and the peak force (N) was recorded.

#### 2.3.4. Water and Oil Absorption Capacity

The method described by Anderson [19] was followed for Water Absorption Capacity (WAC) analysis. One gram of sample was weighed into a 50 mL Falcon tube, and 10 mL of distilled water was added. The mixture was vortexed and incubated in a thermostatic bath at 30 °C for 30 min, with agitation every 5 min. After 30 min, the samples were centrifuged at 4000 rpm for 20 min at 25 °C. The supernatant was carefully decanted, and the tubes were dried on blotting paper for 24 h to remove any residual surface water, thereby obtaining a hydrated sample. The weight of the hydrated sample was recorded, and the WAC was calculated using Equation (3), expressed as the grams of water absorbed relative to the dry sample weight:

(3) $$WAC=\frac{Weight\;of\;water\;absorbed\;\left(g\right)}{Weight\;of\;dry\;sample\;\left(g\right)}$$

where the weight of water absorbed was determined as the difference between the weight of the hydrated sample and the initial dry sample.

The oil absorption capacity (OAC) was determined using the method described by Beuchat [20] with slight modifications. First, 1 g of sample was weighed into a centrifuge tube, and 5 mL of sunflower oil was added. The mixture was vortexed every 5 min for 30 min. The samples were then centrifuged at 4000 rpm for 20 min at 25 °C. The supernatant was carefully discarded, and the tubes were drained on absorbent paper towels to remove excess oil from the walls. Subsequently, the samples were inverted for 24 h to allow for leaching. The final weight of the sample was recorded, and the OAC was calculated using Equation (4), expressed as the grams of oil absorbed relative to the dry sample weight:

(4) $$OAC=\frac{Weight\;of\;oil\;absorbed\;\left(g\right)}{\begin{array}{c} Weight\;of\;dry\;sample \end{array}\;\left(g\right)}$$

where the weight of oil absorbed was determined as the difference between the weight of the oily sample and the initial dry sample.

### 2.4. Chemical Properties

For all chemical analyses described below, the baked tortillas were cooled, freeze-dried, and ground into a fine powder. The water loss during freeze-drying was approximately 41.5%. After that, samples were stored at room temperature in vacuum-sealed bags until analysis.

#### 2.4.1. Proximate Composition

The composition of the tortillas was determined by evaluating the following components: dietary fiber, total sugars, sodium chloride, fat, saturated fatty acids, protein, carbohydrates, moisture, total ash, minerals (calcium, phosphorus, iron, magnesium, potassium, sodium, zinc), and energy value. These components were evaluated using the following methodologies: Fiber content was determined by the official AOAC method 985.29 [21]. Sugar content was determined according to method AOAC [22]. Sodium chloride and sodium were analyzed using the UNE-EN ISO 14,911 methodology [23]. Fat content was determined by Soxhlet extraction in petroleum ether, method 920.39 C [24]. Protein content (Nx6.25) was determined by the Kjeldahl method [25]. Carbohydrates were calculated as the difference between 100 and the sum of protein, fat, moisture, and ash. Moisture and ash content was quantified using gravimetric methods [26,27]. Minerals were evaluated by ICP-MS [28]. Energy content was calculated by summing the energy contributions of fat, carbohydrates, and protein, based on their respective macronutrient composition. The energy values were determined using the Atwater factors: 9 kcal per gram of lipids, 4 kcal per gram of carbohydrates, 4 kcal per gram of protein, and 2 kcal per gram of fiber [29].

#### 2.4.2. Total Polyphenol Content (TPC) and Antioxidant Capacity

Freeze-dried tortilla samples (0.2 g) were mixed with methanol/water (70:30) and processed using ultrasonication followed by vortexing. After centrifugation, the supernatant was filtered and collected. This methanol extract was prepared in quintuplicate. The total polyphenol content (TPC) was measured using a multiscan plate reader (Tecan Infinite M200, Männedorf, Switzerland). Extraction was performed on the same day as the analysis, following the method outlined by Martínez-Sánchez et al. [30] with slight modifications. TPC was measured using the Folin–Ciocalteu method with gallic acid as the standard. In the TPC assay, 20 μL aliquots of the extract were placed into each well of a microplate, followed by 30 μL of the Folin–Ciocalteu reagent (diluted 1:1) and 200 μL of the NaOH/Na_2_CO_3_ mixture. The microplate was then incubated for 45 min at room temperature in the dark. Prior to measurement, the microplate was automatically shaken, and absorbance was measured at 765 nm using a UV-Vis spectrophotometer for the samples and also the calibration curve standards. The TPC was expressed as milligrams of gallic acid equivalent per gram of dry weight (mg GAE/g).

Two methodologies were employed to measure the antioxidant capacity: the ABTS (2,2′-azino-bis (3-ethylbenzothiazoline-6-sulfonic acid)) assay and FRAP (Ferric Reducing Antioxidant Potential) assays. The ABTS assay was conducted following the protocol described by Re et al. [31], whilst the FRAP assay was performed according to the method by Benzie et al. [32], with slight modifications.

The FRAP reagent was prepared by mixing 300 mM sodium acetate (pH 3.6), 10 mM 2,4,6-tripyridyl-s-triazine (TPTZ) in 40 mM hydrochloric acid, and 20 mM ferric chloride solution in a 10:1:1 proportion. Then, 250 μL of that mixture was added to each well of the microplate and incubated for 10 min at 37 °C. The first absorbance reading was taken at 593 nm. Subsequently, 40 μL of the sample extract was added, and the microplate was shaken for 4 min to promote the reaction. A second absorbance reading was then taken at 593 nm. Both assays used Trolox as the calibration standard, and the results were expressed as milligrams of Trolox equivalents per gram of dry weight (mg TE/g).

#### 2.4.3. Identification and Quantification of Glucosinolates

For the identification and quantification of glucosinolates, an Agilent 1200 Liquid Chromatography system (Santa Clara, CA, USA) equipped with a G1311B quaternary pump, G1329B Standard Autosampler, and G1316A column heater was used, coupled with a 6420 triple-quadrupole mass spectrometer (QqQ) with an electrospray ionization (ESI) source. The extraction was carried out following the methodology of Salas-Millán et al. [6], with slight modifications. The freeze-dried samples were diluted in 5 mL of MeOH/H_2_O (70:30). After a hot bath (70 °C), centrifugation, and filtration, 3 mL of the obtained supernatant was dried with nitrogen gas and re-dissolved in 1 or 2 mL of mobile phase A. The mobile phase consisted of A (0.1% formic acid in water) and B (0.1% formic acid in acetonitrile). The autosampler was set to inject 10 μL of the eluted sample at a flow rate of 0.250 mL/min and the gradient was as follows: 5–30% B (14 min), 30–95% B (7 min), and 95–95% B (5 min) at 40 °C. For quantification, the retention time and MS and MS/MS fragmentation spectra were compared with a commercial standard or tentatively identified by comparing their fragmentation patterns with available bibliographic data from the MassBank Europe, MassBank of North America, and PubChem databases. Finally, the glucosinolate results were expressed as µg per 100 g dry weight.

### 2.5. Sensory Analysis

The sensory evaluation was conducted by a trained panel of 12 individuals (7 men and 5 women, aged 21 to 50 years) in Spain. The trained panel consisted of individuals specifically trained to assess products based on defined sensory attributes. The panelists were selected according to their personal taste preferences, tortilla consumption habits, and willingness to purchase tortillas containing broccoli powder. The sensory analysis took place in a controlled testing room equipped with separate booths and water for palate cleansing. Freshly prepared tortillas were evaluated using a subjective rating scale (Table 2). The parameters analyzed included appearance, aroma, taste, texture, and overall acceptability, all rated on a 9-point hedonic scale. The analysis was divided into two phases. In the first phase, baked tortilla samples were preheated, at the same time, in a microwave oven for 10 s and presented to the judges on white plates labeled with random codes. The panelists evaluated the appearance, focusing on tonality and color, as well as the aroma and taste of the tortillas, especially the aroma and taste of corn flour and broccoli. In the second phase, another tortilla was divided in half, preheated, and assessed for texture by considering its bendability and rollability. Bendability was defined as the maximum angle before the tortilla breaks, while rollability was evaluated by rolling the tortilla around a paper straw to observe its performance [33]. Overall acceptability was assessed based on the panelists’ general liking of the tortilla, considering all the sensory attributes mentioned. A score of 5 was considered to be the minimum commercial limit.

This trained sensory evaluation did not require an ethical statement, since it involved no invasive procedures or health-related interventions. Before starting the evaluation, the research team explained the scope and details of the project to the participants, including the purpose of the research, the identity of the researchers, data protection measures, privacy and data retention policies, the voluntary nature of participation, the right to withdraw at any time, and contact details for any questions. Finally, all participants signed a written informed consent form, confirming that they had read and understood the information provided, and that their questions had been answered.

### 2.6. Statistical Analysis

The data obtained were statistically analyzed using a one-way analysis of variance (ANOVA). All parameters were evaluated with a minimum of three replicates and up to a maximum of ten. The results were expressed as mean values ± standard error (SE). Statistical significance was determined at *p* < 0.05, and differences between means were further analyzed using an LSD (Least Significant Difference) test. All statistical analyses were performed using the Statgraphics software (CENTURION XV, 2006).

## 3. Results and Discussion

### 3.1. Physical Properties

#### 3.1.1. Dimensions

The fortified tortillas showed no significant changes in diameter, thickness, or weight compared to the control. For all the treatments, the average diameter of the raw tortillas was 12.9 ± 0.11 cm, while the baked tortillas measured 11.6 ± 0.12 cm. The average thickness remained consistent at 1.6 ± 0.05 mm for both raw and baked samples. Several studies have demonstrated that incorporating ingredients such as pecan shell powder [34] or microalgae [35] (*Nannochloropsis* sp. and *Tetraselmis* sp.) into wheat tortillas does not significantly affect their physical properties, such as thickness and diameter.

#### 3.1.2. Color

The color results for the tortillas are shown in Figure 1 and Table 3. Significant differences were observed in lightness and °h. As the broccoli powder content increased, both lightness and °h consistently decreased. Tortillas made exclusively with corn flour exhibited significantly greater lightness, corresponding to a light corn-yellow color. In contrast, the lowest lightness value was observed in tortillas with 10% of broccoli powder, resulting in a dark olive-green hue. The incorporation of broccoli powder progressively darkened the tortillas, likely due to its naturally darker color (bright lime green) and its high concentrations of pigments such as chlorophyll and carotenoids. Chroma values initially increased with the addition of 2.5% of broccoli powder but decreased progressively as higher amounts of the powder were incorporated. This trend indicated that the color of the tortillas became progressively less vivid and duller as the amount of broccoli powder increased.

Vázquez-Durán et al. [10] also highlighted the impact of vegetable powders on the visual characteristics of fortified food products. Table 4 presents the color properties of broccoli powder, showing a medium lightness, low saturation, and a tone suggesting a yellow-green color. These attributes contributed to the darker and less vivid appearance of the tortillas as the broccoli powder concentration increased. Additionally, this effect was amplified by the high-temperature baking process, which affected the color parameters. These findings are consistent with previous studies, such as the addition of muicle (*Justicia spicigera*) extract into corn tortillas, where thermal processing led to reduced brightness and alterations in °h [36].

#### 3.1.3. Force Test

The firmness of tortillas, as indicated by peak force, decreased significantly with the addition of broccoli powder. As shown in Table 3, the peak strength dropped from 7.08 ± 0.25 N in tortillas made solely with corn flour to 5.09 ± 0.25 in those with a 10% addition of broccoli powder. This drop in firmness indicates a slightly softer firmness for the tortillas with added broccoli powder; this is consistent with the findings of Hassan et al. [37], who observed similar results when enriching tortillas with soybean. Likewise, Rohlfing et al. [38] reported that changes in tortilla composition, such as adding high-amylose corn or resistant starch, also led to significantly decreased firmness due to modifications in structural properties. These results suggest that the inclusion of broccoli powder slightly softens the tortillas, potentially increasing their appeal to a wider range of consumers. The resulting texture, which combines subtle softness with sufficient firmness, may be particularly beneficial for those seeking tortillas that are easier to chew or offer greater pliability, making them ideal for culinary applications such as wraps or tacos, where flexibility is essential.

#### 3.1.4. Water and Oil Absorption Capacity

The incorporation of broccoli by-product powder significantly influenced the water and oil absorption capacities of the tortillas (Table 3). As the percentage of broccoli powder was increased, both WAC and OAC showed progressive rises. Specifically, tortillas with 10% broccoli powder exhibited the highest absorption capacities, with WAC increasing from 2.13 ± 0.04 g/g in the control to 2.57 ± 0.04 g/g, and OAC rising from 0.71 ± 0.02 g/g to 0.89 ± 0.02 g/g.

The increase in WAC is linked to the hydrophilic nature of dietary fibers, particularly cellulose and hemicellulose, which enhance water retention and swelling capacity [33,39]. Similarly, the higher OAC can be attributed to the fibrous and porous structure of the added ingredients, which promotes oil retention through hydrophobic interactions within the fiber matrix [40] and could influence the mouthfeel profile. Singh et al. [41] demonstrated, for instance, that freeze-dried corn gluten meals with a porous structure exhibited improved water and oil retention. Additionally, the inclusion of fiber-rich ingredients not only enhances absorption properties but also improves texture and stability during processing, making such ingredients valuable for food applications like the corn tortillas in the present study [42].

### 3.2. Chemical Properties

#### 3.2.1. Proximate Composition

The addition of broccoli by-product powder significantly enhanced the mineral content of tortillas (Table 5). The calcium and potassium content increased most, with calcium rising from 210 mg/kg in control to 1213.5 mg/kg at 10% of broccoli powder, a nearly six-fold increase, whilst potassium increased from 2443.5 mg/kg to 7582.5 mg/kg, a threefold rise. Magnesium, sodium, and phosphorus also showed notable improvements, with increases of 1.4-, 1.1-, and 1.1-fold, respectively, at the highest level of fortification. Among the microminerals, iron and zinc concentrations increased significantly, with iron reaching 38 mg/kg and zinc 15 mg/kg at 10% of broccoli powder, corresponding to 3-fold and 1.36-fold enhancements. These findings align with studies which demonstrated that incorporating nutrient-dense flours, such as broccoli or other vegetable powders, improves the nutritional profile of corn-based products [10]. Similar fortification efforts using alternative ingredients, such as *Moringa oleifera* flour [43] or carotenoid-rich flour substitutes [44], further support the potential of such approaches. This positions tortillas fortified with broccoli by-product as a promising vehicle to enhance both macro- and micronutrient intake in staple diets. The current inadequate intake of nutrient-dense foods and beverages across food groups has resulted in the underconsumption of certain nutrients and dietary components. Calcium, potassium, dietary fiber, and vitamin D are identified as nutrients of public health concern for the general U.S. population given that their low consumption has been linked to health issues [45].

Calcium is essential for bone health, muscle function, and nerve signaling. Adequate calcium intake throughout life is crucial for maintaining bone density and preventing osteoporosis [46]. In general, the main sources of calcium for humans are dairy products such as milk and cheese. Milk is one of the richest sources of calcium, providing approximately 1200 mg per liter [47]. Regarding potassium, its intake is essential for maintaining various physiological functions, including blood pressure regulation, nerve transmission, and muscle contraction.

The National Academy of Medicine recommends a potassium intake for adults of 3400 mg per day for men and 2600 mg per day for women [48]. Consuming potassium-rich foods can help meet these daily requirements. Spinach, bananas, and white beans are valued for their richness in potassium, with concentrations that range from 3226 to 5820 mg/kg (fresh weight). Iron is a crucial mineral required for the synthesis of hemoglobin, the protein responsible for transporting oxygen throughout the body. An iron deficiency can result in anemia, which manifests as fatigue, weakness, and impaired cognitive function [49]. Certain populations are more susceptible to iron deficiency and may require supplementation to meet their needs. The required iron dose varies depending on individual health conditions and specific needs. For instance, adults diagnosed with iron deficiency anemia are commonly prescribed between 150 and 200 mg of elemental iron daily [50]. Dietary iron exists in two forms: heme and non-heme. Heme iron, found in animal-based foods such as lean meats, poultry, and seafood, is absorbed more efficiently by the body compared to non-heme iron, which is present in plant-based foods like legumes, lentils, and leafy greens. In addition to these natural sources, iron-fortified foods, such as whole-wheat bread and ready-to-eat cereals, can also provide a major contribution to daily iron intake. However, the effectiveness of iron fortification depends not only on the total iron content but also on its bioavailability. Considering this, while the fortified tortillas analyzed in this study may have increased iron content, it is necessary to assess their bioaccessibility and the potential impact on human iron status.

It is important to highlight that in our study, the mineral content is expressed per kilogram of dry weight (freeze-dried tortillas). However, in practice, according to data from the National Council for the Evaluation of Social Development Policy, the per capita consumption of tortillas in Mexico is approximately 56.7 kg per year, equivalent to around 155 g per day in fresh weight. Considering this amount, the mineral intake from these tortillas does not exceed the limits established by the Recommended Dietary Allowance.

The dietary fiber increased significantly with higher levels of fortification, ranging from 6.70 g/100 g in the control group to 8.25 g/100 g at 10% fortification, with the 7.5% fortification showing a statistically significant increase compared to the control. Broccoli is a vegetable that provides a significant amount of dietary fiber, mostly insoluble fiber; for example, fresh and raw broccoli provides approximately 2.4 g of fiber per 100 g [51]. Insoluble fiber promotes regular bowel movements, supports gut health by feeding beneficial bacteria, and may reduce the risk of conditions such as diverticulitis and colorectal cancer [52]. In today’s population, where diets are often high in ultra-processed foods and low in fiber, it is important to improve overall wellbeing. Santamaria et al. [53] found that incorporating cabbage varieties, particularly pak choi, into flatbread formulations significantly increased total dietary fiber content compared to the control, thus highlighting the potential of the *Brassica* genus as a source of fiber.

The protein content also showed an upward trend, from 7.45 g/100 g in the control to 8.65 g/100 g in the 10% sample, becoming significant with fortification starting at 5%. However, the protein content was low, and this product is not particularly important for increasing protein consumption; to achieve this, it is recommended to consume high-protein foods such as lean meats, fish, eggs, legumes, dairy, and plant-based sources like tofu and quinoa.

Carbohydrate levels showed a slight reduction at higher fortification levels. Tortillas fortified with 10% of broccoli powder contained 71.10 g/100 g of carbohydrates compared to 73.95 g/100 g in the control. This reduction can be attributed to the replacement of corn flour, which is primarily composed of starch—a complex carbohydrate that provides a high amount of digestible carbohydrates—with broccoli powder. Broccoli powder, on the other hand, has a much lower carbohydrate content and contains a substantial amount of insoluble fiber, which does not contribute to the digestible carbohydrate fraction.

The total lipid content exhibited a comparable trend to the carbohydrate content, with a slight reduction observed at higher fortification levels. Tortillas with 10% fortification contained 2.60 g/100 g, compared to 2.80 g/100 g in the control. Given that broccoli powder is a low-fat ingredient, the replacement of corn flour with broccoli resulted in a reduction in the overall fat content of the tortillas. However, that change was minimal, as the proportion of broccoli powder used in the substitution is likely to be low. Consequently, no significant differences were observed in the saturated fatty acid levels among the treatments.

The effect of fortification with broccoli powder was evidenced by an increase in the total sugar content of the samples, with values of 1.3 g/100 g in the control and 2.7 g/100 g at a 10% substitution level. According to the United States Department of Agriculture (USDA), fresh and raw broccoli contains approximately 1.4 g total sugars per 100 g fresh weight [51], while corn flour, widely used as a base ingredient in many food formulations, has a lower sugar content of around 1.04 g total sugars per 100 g [54]. This highlights that broccoli has a higher proportion of simple sugars, such as glucose, fructose, and sucrose, whereas corn flour consists primarily of starches, which are not classified as simple sugars in conventional carbohydrate analyses. Furthermore, during the drying and freeze-drying processes used to produce broccoli powder, water is removed, concentrating the sugar content and increasing its proportion in terms of dry weight. This effect, combined with broccoli’s naturally higher sugar content compared to corn flour, explains the observed increase.

The ash content, which indicates the total mineral presence, showed a steady rise from 3.2 g/100 g in the control to 4.35 g/100 g in tortillas fortified with 10% broccoli powder, which is to be expected after a significant increase in macro- and microminerals by using the broccoli powder.

The sodium chloride (salt) content showed a slight increase in the fortified tortillas, with significant differences observed among treatments. The control sample contained 2.66 g/100 g, while the 10% fortified tortillas had 2.96 g/100 g. It should be recalled that the proximal composition evaluation was performed in freeze-dried tortillas, where water removal concentrated the nutrients, including salt. As a result, the actual salt content in fresh tortillas would be lower. This minor increase in salt is unlikely to pose health risks when tortillas are consumed in moderation. Salt is known to play an essential role in various physiological functions, such as maintaining fluid balance and supporting nerve signaling. However, excessive salt intake has been linked to health issues, including high blood pressure, cardiovascular disease, and kidney problems. While the increase in sodium chloride from fortification is minimal, it is important to consider total sodium intake from all dietary sources to ensure that recommended limits are not exceeded [55].

The residual moisture content in freeze-dried tortillas decreased as the proportion of broccoli powder increased, dropping from 6.65 g/100 g in the control to 4.75 g/100 g in the tortillas fortified at 10%, which could indeed be attributed to the higher fiber content. As mentioned before, broccoli, since it is rich in fiber (especially insoluble fiber), has a higher ability to bind water compared to corn flour, leading to a lower overall moisture content in the final product. This effect has been previously observed in fortified tortillas, where the higher fiber content resulted in greater WAC. The reduction in moisture content might also contribute to extended shelf life.

The energy value of fortified tortillas was slightly lower compared to the control tortillas (364.2 kcal/100 g), primarily due to a modest reduction in carbohydrates and lipids, particularly in formulations with higher percentages of broccoli powder replacing corn flour. From the perspective of proximate composition, these fortified tortillas offer notable health benefits, especially in addressing dietary deficiencies in key minerals such as calcium, potassium, and iron, as well as dietary fiber.

#### 3.2.2. TPC and Antioxidant Capacity

Tortillas fortified with broccoli by-product powder exhibited a remarkable enhancement in TPC and antioxidant activity (Table 6).

The control tortillas had a TPC of 0.87 ± 0.02 mg GAE/g. The addition of broccoli significantly increased the TPC, reaching 2.30 ± 0.04 mg GAE/g at the 10% fortification level, a 2.6-fold increase compared to the control. The incorporation of broccoli by-products such as stems and leaves can effectively enrich food matrices due to their high content of polyphenols and other bioactive compounds [11,56]. The antioxidant capacity, evaluated through ABTS and FRAP assays, revealed similar trends. The ABTS showed a value of 0.61 ± 0.03 mg TE/g for the control treatment, which increased significantly across all fortification levels, reaching 0.99 ± 0.02 mg TE/g with 10% of broccoli powder. This progressive increase underscores the contribution of broccoli-derived antioxidants to the total antioxidant activity of fortified tortillas. Similarly, the FRAP assay confirmed said antioxidant trend but required at least 5% of broccoli powder to achieve statistically significant differences compared to the control. At 10% fortification, FRAP values peaked at 0.29 ± 0.01 mg TE/g, almost double the control level of 0.15 ± 0.01 mg TE/g. These results are consistent with other studies on the incorporation of broccoli by-products to enhance the functional composition of foods. For instance, Krupa-Kozak et al. [57] demonstrated that gluten-free bread enriched with broccoli leaf powder exhibited significantly higher TPC and antioxidant activity. Similarly, Fanesi et al. [13] incorporated broccoli-derived extracts into bakery products, enhancing the levels of bioactive compounds, including polyphenols and glucosinolates, preserving their functionality in the food matrix. Furthermore, a study by Uuh Narvaez et al. [58] demonstrated that adding cruciferous vegetable by-products, such as cabbage, to corn tortillas enhanced their functional and antioxidant properties, highlighting their potential as a dietary option for diabetes management and supporting the feasibility of fortifying staple foods with such ingredients. The antioxidant capacity of the fortified tortillas can be directly linked to the bioactive profile of the broccoli powder used as an ingredient. The broccoli powder analyzed (Table 4) had 9.07 mg GAE/g of TPC, coupled with significant antioxidant activity measured by the ABTS (6.66 mg TE/g) and FRAP (3.06 mg TE/g) assays. Numerous studies have demonstrated that broccoli by-products contain a variety of compounds with substantial antioxidant capacity [6,59,60,61]. The main function of antioxidants is to slow the oxidation of other molecules by inhibiting free radical chain reactions, thereby reducing oxidative damage in the human body. Such oxidative damage can contribute significantly to the development of chronic diseases, including cancer and cardiovascular disease [62].

#### 3.2.3. Identification and Quantification of Glucosinolates

**Figure 2:**
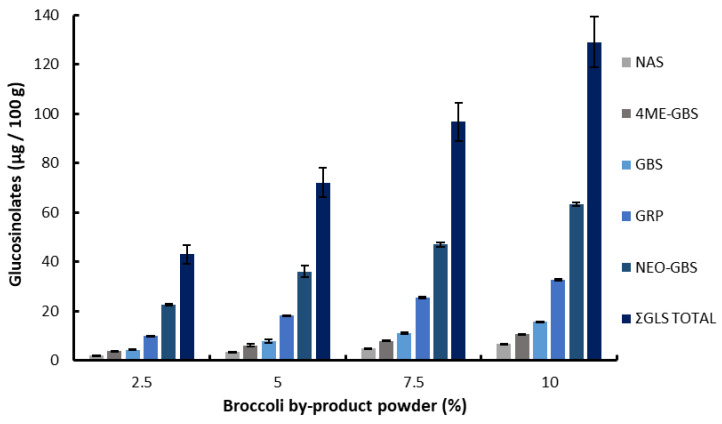
Glucosinolates (GLSs) in cooked tortillas fortified at different levels of broccoli by-product powder. NEO-GBS: Neoglucobrassicin; GRP: glucoraphanin; GBS: glucobrassicin; 4ME-GBS: 4-Methoxyglucobrassicin; NAS: gluconasturtiin. Mean values (*n* = 5) ± standard errors. Data expressed on a dry weight basis (freeze-dried tortillas).

The identification and quantification of glucosinolates in both broccoli powder and fortified tortillas are presented in Table 4 and Figure 2, respectively. Neoglucobrassicin, glucoraphanin, glucobrassicin, 4-methoxyglucobrassicin, gluconasturtiin, glucotropaeolin, and glucoerucin were identified in the broccoli by-product powder. According to Liu et al. [59], glucoraphanin and neoglucobrassicin are the main glucosinolates found in broccoli tissues, with significant concentrations not only in florets but also in stems and leaves. Processing methods, such as thermal treatments, play a crucial role in preserving these compounds [63]; therefore, the drying process for obtaining broccoli powder is crucial. To that end, stalks and leaves were dried under hot air at temperatures between 45 °C and 75 °C.

It should be noted that glucotropaeolin and glucoerucin were not detected in the fortified tortillas; this may be attributed to low concentrations in the broccoli powder, so there might not be enough of these two glucosinolates to be detectable. In contrast, the remaining glucosinolates were well preserved during the baking process and actually increased with the percentage of broccoli powder incorporated into the formulation (Figure 2). Key glucosinolates, such as neoglucobrassicin and glucoraphanin, demonstrated excellent retention during the baking process, with concentrations closely matching the expected values based on the percentage of broccoli powder incorporated, highlighting their remarkable stability under the thermal conditions of baking at 300 °C for 6 min. This is consistent with the findings of Ares et al. [64] and Jones et al. [65], who reported that the stability of glucosinolates depends on the plant type, initial concentration, amount of water, thermal controlled drying, and storage conditions and can effectively preserve those compounds even after thermal treatment. The tortilla baking process did not lead to significant glucosinolate losses, as demonstrated by the strong correspondence between the glucosinolate content in the fortified tortillas and the values predicted based on the original broccoli powder concentrations. Nartea et al. [14] observed that glucosinolate concentrations rose proportionally with higher cauliflower by-product fortification, a trend consistent with our broccoli-fortified tortillas. In our experiment, that pattern was consistent across all glucosinolate compounds, highlighting the stability of these bioactive components during the baking process, which emphasizes the efficacy of this fortification strategy. This was consistent with the stability observed in other broccoli-based products [6,14,66], thereby indicating the potential for substantial nutritional benefits associated with higher fortification levels. As a potential health benefit, the presence of glucosinolates, primarily found in cruciferous vegetables, provides a key source of anticarcinogenic compounds. Glucosinolates from the indolic group, including glucobrassicin, neoglucobrassicin, and 4-methoxyglucobrassicin, are hydrolyzed to produce bioactive derivatives such as indole-3-carbinol [67].

This compound is particularly known for its potent antioxidant, anticancer, and anti-inflammatory properties. It has been shown to modulate the metabolism of estrogen, inhibit tumor growth, and reduce the risk of hormone-dependent cancers. Non-indolic glucosinolates such as glucoraphanin and gluconasturtiin contribute to health benefits through other mechanisms. These glucosinolates are hydrolyzed into sulfur-containing bioactive compounds such as sulforaphane (from glucoraphanin) which exhibit strong antioxidant and detoxifying properties. These compounds help protect against oxidative stress, a key factor in chronic diseases such as cancer and cardiovascular conditions, while supporting cellular detoxification and promoting overall health [68].

### 3.3. Sensory Analysis

The results of the sensory analysis are presented in Table 7. The appearance of the tortillas was evaluated by visual tonality and color acceptability. Tonality increased significantly with higher percentages of broccoli by-product powder, from 1.08 ± 0.08 in the control treatment to 6.5 ± 0.29 in tortillas fortified with 10% of broccoli powder. This increase corresponds with the color parameters detailed in Table 3, where the color shifted from pale to a dark olive-green hue as fortification levels increased. This change in color was positively associated with higher acceptability scores, suggesting that fortified tortillas were preferred over the control pale yellow corn tortilla, particularly at elevated fortification levels. For the aroma attribute, both corn and brassica aromas were evaluated, as well as aroma acceptability. The panelists reported a decline in corn aroma intensity with increasing fortification levels; the scores decreased from 6.08 ± 0.77 in the control to 1.75 ± 0.25 in the tortillas fortified with 10% of broccoli by-products. This finding suggested that the distinctive corn aroma was overshadowed by the presence of broccoli by-products, resulting in an augmentation in the prevailing brassica aroma as fortification levels increased. Consequently, the highest levels of aroma acceptability were observed for the control and the tortillas fortified with 2.5% and 5% of broccoli powder, respectively. However, a significant decrease in aroma acceptability was recorded at the 10% fortification level, primarily due to the pronounced brassica aroma, which was not well accepted by the panelists.

The evaluation of the taste parameter was conducted in a manner analogous to that of the aroma, focusing on both corn and brassica taste, as well as overall taste acceptability. The corn taste declined substantially with increasing fortification levels, dropping from 7.25 ± 0.43 in the control treatment to 2.08 ± 0.40 in tortillas fortified with 10% of broccoli by-product powder. The characteristic corn taste was overshadowed by the taste from the broccoli by-products. In fact, the brassica taste became more pronounced with higher levels of fortification. The highest level of acceptability for taste was at 2.5% fortification (7.50 ± 0.23), with the lowest at 10% (5.00 ± 0.35), indicating that an increase in the percentage of broccoli by-products negatively affected taste acceptability. For the texture evaluation, both consistency and rollability were assessed. No significant differences were found among the treatments, suggesting that fortification with broccoli by-products did not exert a detrimental effect on those parameters. This finding is of particular significance, given that a tortilla must possess the appropriate texture to adequately contain various fillings and demonstrate sufficient rollability for the effective preparation of tacos and analogous dishes. Maintaining these textural qualities is crucial for ensuring that the fortified tortillas remain functional and appealing to consumers, enhancing their potential acceptance in culinary applications.

Finally, the panelists assessed the overall acceptability; the fortified tortillas that received the highest scores were those that had been fortified at 2.5%, 5%, and 7.5% (ranging from 6.58 to 6.33). In contrast, the control and the tortillas that had been fortified at 10% received the lowest scores, 5.67 and 5.00, respectively. These results indicate that while moderate fortification was well accepted, higher levels of fortification negatively impacted the overall acceptability of the tortillas. This suggests a need to balance nutritional enhancement with sensory qualities to optimize consumer acceptance. It is important to note that the overall acceptability score is based on a comparative evaluation of all the tortilla samples against each other. Additionally, tortillas are traditionally consumed with other ingredients rather than on their own. In our sensory analysis, trained panelists evaluated the tortillas without any fillings to avoid potential influences from added ingredients. This methodological choice may explain the generally lower scores observed across all treatments, including the control, and should not be interpreted as a sign of reduced sensory quality. Therefore, in terms of sensory parameters, fortification of tortillas at concentrations between 2.5% and 7.5% appeared to be optimal for overall acceptability, whilst the 10% fortification was the least accepted by consumers despite being rated above five, which was the commercial limit. Previous studies on the sensory evaluation of tortillas have reported a similar trend, where increased concentrations correlated with decreased consumer acceptability. For example, Artavia et al. [44] found that replacing part of the corn flour with non-traditional flours rich in carotenoids (such as sweet potato, peach palm, and cassava) resulted in lower acceptability ratings for tortillas with higher concentrations of these flours. Additionally, Vázquez-Durán et al. [10] added broccoli powder to chips, reporting that products with an elevated broccoli content were often rejected by consumers, primarily due to stronger flavors and aromas. In contrast, Hernández-López et al. [35] fortified tortillas with marine microalgae, suggesting that consumer acceptance could be improved by informing them of the nutritional benefits, thereby positioning these products favorably within the health-conscious and sustainable food markets. However, the sale of products made from by-products may be poorly received by some consumers, either because of a perception of lower quality or a lack of awareness about the origin of these materials. This perception could influence them to avoid the consumption of such products [8], highlighting the importance of consumer education in this area.

## 4. Conclusions

This study evaluated the effect on the physical, chemical, and sensory properties of corn tortillas fortified with different concentrations of broccoli by-product powder. The results showed that the addition of broccoli powder did not significantly alter the basic dimensions of the tortillas, with their structural integrity being maintained. However, fortification did affect the color, which changed from light corn-yellow to dark olive-green tones, particularly at higher fortification levels. The slight increase in WAC and OAC in fortified tortillas suggests that the broccoli fiber enhances moisture and oil retention, improving their culinary appeal. On the other hand, the firmness of the tortillas decreased slightly with increasing broccoli powder concentration, indicating a very slight softening effect which may be of interest to certain consumers.

Nutritionally, fortification with broccoli by-product powder significantly improved the profile of the tortillas, increasing concentrations of essential minerals such as calcium (six-fold) and potassium and iron (three-fold), as well as the fiber content. The antioxidant capacity also improved substantially, with the total polyphenol content (TPC) exhibiting a 2.6-fold increase compared to the control. Among glucosinolates, neoglucobrassicin and glucoraphanin were present at the highest concentrations, followed by glucobrassicin, 4-methoxyglucobrassicin, and gluconasturtiin, demonstrating their stability despite the baking process applied to the tortillas. Sensory evaluation revealed that fortification levels of between 2.5% and 7.5% were well accepted, with positive feedback regarding color, aroma, and taste. Overall, fortification of corn tortillas with broccoli by-products represents a promising strategy for improving the nutritional profile of this staple food, promoting healthier diets while reducing food waste and supporting public health.

## Figures and Tables

**Table 1:** Composition (%) of control and fortified tortillas with varying percentages of broccoli by-product powder.

Ingredients	Control	Tortillas Fortified with Broccoli By-Product Powder (%)			
		2.5%	5%	7.5%	10%
Water (mL)	59	59	59	59	59
Corn flour (g)	40	37.5	35	32.5	30
Broccoli powder (g)	0	2.5	5	7.5	10
Salt (g)	1	1	1	1	1

**Table 2:** Descriptive sensory evaluation of tortillas.

Parameters		Score
Appearance	Tonality	1 = very light, 5 = intermediate, 9 = very dark
	Color acceptability	1 = dislike extremely, 5 = neither dislike nor like, 9 = like extremely
Aroma and Taste	Corn or brassica aroma/taste	1 = extremely weak, 5 = neither intense nor weak, 9 = extremely intense
	Aroma/Taste acceptability	1 = dislike extremely, 5 = neither dislike nor like, 9 = like extremely
Texture	Bending	1 = none, 3 = 15°, 5 = 45°, 7 = 60°, 9 = 90°
	Rollability	1 = does not roll, 4 = breaks between the two sides, 6 = breaks on one side, 9 = rolls without breaking
Overall acceptability		1 = dislike extremely, 5 = neither dislike nor like, 9 = like extremely

**Table 3:** Physical properties of baked control corn tortillas and corn tortillas fortified with broccoli by-product powder.

Properties	* Control		Broccoli By-Product Powder (%)			
			2.5%	5%	7.5%	10%
Color						
L*	73.93 ± 0.27 ^z a^		58.92 ± 0.25 ^b^	50.07 ± 0.32 ^c^	46.51 ± 0.14 ^d^	43.15 ± 0.23 ^e^
°h	94.57 ± 0.07 ^a^		84.53 ± 0.14 ^b^	80.43 ± 0.17 ^c^	79.20 ± 0.12 ^d^	77.57 ± 0.27 ^e^
C*	15.58 ± 0.17 ^d^		17.90 ± 0.07 ^a^	17.32 ± 0.09 ^b^	16.34 ± 0.05 ^c^	15.41 ± 0.07 ^d^
Firmness						
Peak force (N)	7.08 ± 0.25 ^a^		6.46 ± 0.13 ^b^	5.41 ± 0.13 ^c^	5.38 ± 0.21 ^c^	5.09 ± 0.25 ^c^
Absorption Capacity						
Water		2.13 ± 0.04 ^c^	2.37 ± 0.03 ^b^	2.48 ± 0.03 ^ab^	2.56 ± 0.05 ^a^	2.57 ± 0.04 ^a^
Oil		0.71 ± 0.02 ^c^	0.78 ± 0.04 ^b^	0.86 ± 0.04 ^a^	0.87 ± 0.02 ^a^	0.89 ± 0.02 ^a^

^z^ Mean values (*n* = 10) ± standard errors for color (*n* = 5), firmness, and absorption capacity. For each parameter, different letters denote statistically significant differences among treatments (*p* < 0.05). * Control: Tortilla formulated with 100% corn flour.

**Table 4:** Color, total phenolic content (TPC), antioxidant capacity (ABTS and FRAP), and glucosinolates content of broccoli by-product powder.

Properties		Broccoli Powder
Color	L*	60.89 ± 0.15 ^z^
	°h	83.36 ± 0.05
	C*	21.10 ± 0.04
TPC (mg GA/g)		9.07 ± 0.17
ABTS (mg TE/g)		6.66 ± 0.10
FRAP (mg TE/g)		3.06 ± 0.18
Glucosinolates (µg/100 g)	Neoglucobrassicin	578.01 ± 14.95
	Glucoraphanin	211.99 ± 2.02
	Glucobrassicin	166.88 ± 5.34
	4-Methoxyglucobrassicin	80.80 ± 2.15
	Gluconasturtiin	59.46 ± 0.81
	Glucotrapeolin	2.38 ± 0.01
	Glucoerucin	2.08 ± 0.02

^z^ Mean values (*n* = 5) ± standard error. All results are expressed on a dry weight basis. TE (Trolox equivalent) and GA (gallic acid equivalent).

**Table 5:** Proximate composition of baked control corn tortillas and corn tortillas fortified with broccoli by-product powder.

Properties	* Control	Broccoli By-Product Powder (%)			
		2.5%	5%	7.5%	10%
Sodium (mg/kg)	10,637.0 ± 2.0 ^z c^	11,027.5 ± 66.5 ^b^	11,230.5 ± 40.5 ^b^	11,590.5 ± 57.5 ^a^	11,799.0 ± 35.0 ^a^
Potassium (mg/kg)	2443.5 ± 28.5 ^e^	3711.5 ± 36.5 ^d^	4959.5 ± 41.5 ^c^	6348 ± 18.0 ^b^	7582.5 ± 66.5 ^a^
Magnesium (mg/kg)	774.5 ± 5.5 ^e^	852.5 ± 8.5 ^d^	916.0 ± 4.0 ^c^	1018.0 ± 3.0 ^b^	1094.5 ± 4.5 ^a^
Calcium (mg/kg)	210.0 ± 4.0 ^e^	462.5 ± 2.5 ^d^	714.5 ± 6.5 ^c^	986.5 ± 5.5 ^b^	1213.5 ± 4.5 ^a^
Phosphorus (mg/100 g)	192.8 ± 4.2 ^b^	197.9 ± 3.6 ^b^	201.5 ± 4.7 ^ab^	206.5 ± 4.8 ^ab^	213.7 ± 0.1 ^a^
Iron (mg/kg)	12.5 ± 0.5 ^e^	20.0 ± 0.1 ^d^	25.5 ± 0.5 ^c^	33.0 ± 1.0 ^b^	38.0 ± 1.0 ^a^
Zinc (mg/kg)	11.00 ± 0.01 ^d^	12.75 ± 0.25 ^c^	13.00 ± 0.01 ^c^	14.00 ± 0.01 ^b^	15.00 ± 0.01 ^a^
Dietary fiber (g/100 g)	6.70 ± 0.10 ^b^	7.05 ± 0.15 ^ab^	7.25 ± 0.25 ^ab^	7.65 ± 0.25 ^a^	8.25 ± 0.05 ^a^
Protein (g/100 g)	7.45 ± 0.15 ^c^	7.75 ± 0.05 ^bc^	8.10 ± 0.10 ^ab^	8.45 ± 0.15 ^a^	8.65 ± 0.25 ^a^
Carbohydrates (g/100 g)	73.95 ± 0.05 ^a^	72.65 ± 0.35 ^ab^	71.90 ± 0.60 ^b^	71.70 ± 0.70 ^b^	71.10 ± 0.10 ^b^
Lipids (g/100 g)	2.80 ± 0.01 ^a^	2.70 ± 0.01 ^b^	2.60 ± 0.20 ^c^	2.60 ± 0.10 ^c^	2.60 ± 0.02 ^c^
Saturated fatty acids (g/100 g)	0.45 ± 0.05 ^ns^	0.45 ± 0.05 ^ns^	0.45 ± 0.05 ^ns^	0.45 ± 0.01 ^ns^	0.45 ± 0.01 ^ns^
Total sugars (g/100 g)	1.30 ± 0.01 ^e^	1.55 ± 0.05 ^d^	2.05 ± 0.05 ^c^	2.35 ± 0.05 ^b^	2.70 ± 0.01 ^a^
Total ash (g/100 g)	3.20 ± 0.01 ^e^	3.55 ± 0.05 ^d^	3.85 ± 0.05 ^c^	4.15 ± 0.05 ^b^	4.35 ± 0.05 ^a^
Sodium chloride (g/100 g)	2.66 ± 0.01 ^e^	2.75 ± 0.01 ^d^	2.82 ± 0.12 ^c^	2.89 ± 0.03 ^b^	2.96 ± 0.01 ^a^
Moisture content (g/100 g)	6.65 ± 0.05 ^a^	6.50 ± 0.2 ^a^	5.35 ± 0.05 ^b^	5.35 ± 0.15 ^b^	4.75 ± 0.35 ^b^
Energy value (kcal/100 g)	364.2 ± 0.6 ^a^	360.4 ± 0.7 ^b^	358.4 ± 1.4 ^b^	360.2 ± 1.4 ^b^	361.6 ± 1.3 ^b^

^z^ Mean value (*n* = 3) ± standard errors. Data are expressed on a dry weight basis (freeze-dried tortillas). For each parameter, different letters denote statistically significant differences among treatments (*p* < 0.05). ^ns^: No significant differences. * Control: Tortilla formulated with 100% corn flour.

**Table 6:** Total phenolic compounds (TPCs) and antioxidant capacity (ABTS, FRAP) of baked control corn tortillas and corn tortillas fortified with broccoli by-product powder.

Properties	* Control	Broccoli By-Product Powder (%)			
		2.5%	5%	7.5%	10%
TPC (mg GA/g)	0.87 ± 0.02 ^z e^	1.11 ± 0.03 ^d^	1.30 ± 0.05 ^c^	1.79 ± 0.06 ^b^	2.30 ± 0.04 ^a^
ABTS (mg TE/g)	0.61 ± 0.03 ^d^	0.65 ± 0.03 ^c^	0.68 ± 0.02 ^c^	0.83 ± 0.04 ^b^	0.99 ± 0.02 ^a^
FRAP (mg TE/g)	0.15 ± 0.01 ^c^	0.17 ± 0.01 ^c^	0.21 ± 0.01 ^b^	0.25 ± 0.02 ^b^	0.29 ± 0.01 ^a^

^z^ Mean (*n* = 5 ± standard errors). For each parameter, different letters denote statistically significant differences among treatments (*p* < 0.05). * Control: Tortilla formulated with 100% corn flour. TE (Trolox equivalent) and GA (gallic acid equivalent) per dry weight (freeze-dried tortillas).

**Table 7:** Sensory evaluation of baked control corn tortillas and corn tortillas fortified with broccoli by-product powder.

Attributes		Control *	Broccoli By-Product Powder (%)			
			2.5%	5%	7.5%	10%
Appearance	Visual tonality	1.08 ± 0.08 ^z e^	3.75 ± 0.28 ^d^	5.58 ± 0.19 ^c^	6.50 ± 0.29 ^b^	7.50 ± 0.29 ^a^
	Color acceptability	6.08 ± 0.73 ^c^	6.85 ± 0.51 ^bc^	7.17 ± 0.46 ^b^	8.60 ± 0.19 ^a^	8.58 ± 0.19 ^a^
Aroma	Corn aroma	6.08 ± 0.77 ^a^	4.33 ± 0.58 ^b^	3.50 ± 0.55 ^bc^	2.50 ± 0.44 ^cd^	1.75 ± 0.25 ^d^
	Brassica aroma	1.17 ± 0.12 ^d^	3.75 ± 0.55 ^c^	5.58 ±0.59 ^b^	6.17± 0.51 ^b^	7.75 ± 0.46 ^a^
	Aroma acceptability	7.10 ± 0.33 ^ab^	7.5 ± 0.33 ^a^	7.33 ± 0.33 ^a^	6.33 ± 0.43 ^bc^	5.50 ± 0.33 ^c^
Taste	Corn taste	7.25 ± 0.43 ^a^	5.50 ± 0.36 ^b^	4.00 ± 0.49 ^c^	3.00 ± 0.46 ^c^	2.08 ± 0.40 ^d^
	Brassica taste	1.08 ± 0.08 ^d^	4.58 ± 0.57 ^c^	4.75 ± 0.59 ^bc^	6.25 ± 0.55 ^b^	7.83 ± 0.65 ^a^
	Taste acceptability	6.58 ± 0.23 ^bc^	7.50 ± 0.23 ^a^	6.67 ± 0.35 ^ab^	5.75 ± 0.30 ^cd^	5.00 ± 0.35 ^d^
Texture	Bending	7.67 ± 0.14 ^ns^	7.67 ± 0.23 ^ns^	7.68 ± 0.50 ^ns^	7.50 ± 0.60 ^ns^	7.75 ± 0.57 ^ns^
	Rollability	8.08 ± 0.31 ^ns^	8.25 ± 0.18 ^ns^	8.09 ± 0.25 ^ns^	8.33 ± 0.30 ^ns^	8.25 ± 0.22 ^ns^
Overall acceptability		5.67 ± 0.61 ^b^	6.58 ± 0.36 ^a^	6.42 ± 0.61 ^a^	6.33 ± 0.60 ^a^	5.00 ± 0.73 ^b^

^z^ Mean (*n* = 12 ± standard errors). For each parameter, different letters denote statistically significant differences among treatments (*p* < 0.05). ^ns^: No significant differences. * Control: Tortilla formulated with 100% corn flour.

## Data Availability

The original contributions presented in this study are included in the article. Further inquiries can be directed to the corresponding author.
